# A Lipidomic Analysis of Docosahexaenoic Acid (22:6, ω3) Mediated Attenuation of Western Diet Induced Nonalcoholic Steatohepatitis in Male *Ldlr ^-/-^* Mice

**DOI:** 10.3390/metabo9110252

**Published:** 2019-10-28

**Authors:** Manuel García-Jaramillo, Kelli A. Lytle, Melinda H. Spooner, Donald B. Jump

**Affiliations:** 1 Nutrition Program, School of Biological and Population Health Sciences, Oregon State University, Corvallis, OR 97331, USA; Manuel.g.jaramillo@oregonstate.edu (M.G.-J.); Kelli.Lytle@mayo.edu (K.A.L.); Spoonerm@oregonstate.edu (M.H.S.); 2 Department of Chemistry, Oregon State University, Corvallis, OR 97331, USA; 3 The Linus Pauling Institute, Oregon State University, Corvallis, OR 97331, USA

**Keywords:** nonalcoholic fatty liver disease, nonalcoholic steatohepatitis, arachidonic acid, docosahexaenoic acid, inflammation, fibrosis, lipidomics, mass spectrometry

## Abstract

Nonalcoholic fatty liver disease (NAFLD) is a major public health problem worldwide. NAFLD ranges in severity from benign steatosis to nonalcoholic steatohepatitis (NASH), cirrhosis, and primary hepatocellular cancer (HCC). Obesity and type 2 diabetes mellitus (T2DM) are strongly associated with NAFLD, and the western diet (WD) is a major contributor to the onset and progression of these chronic diseases. Our aim was to use a lipidomic approach to identify potential lipid mediators of diet-induced NASH. We previously used a preclinical mouse (low density lipoprotein receptor null mouse, *Ldlr ^-/-^)* model to assess transcriptomic mechanisms linked to WD-induced NASH and docosahexaenoic acid (DHA, 22:6, ω3)-mediated remission of NASH. This report used livers from the previous study to carry out ultra-high-performance liquid chromatography coupled with tandem mass spectrometry (LC-MS/MS) and high-performance liquid chromatography coupled with dynamic multi-reaction monitoring (HPLC-dMRM) to assess the impact of the WD and DHA on hepatic membrane lipid and oxylipin composition, respectively. Feeding mice the WD increased hepatic saturated and monounsaturated fatty acids and arachidonic acid (ARA, 20:4, ω6) in membrane lipids and suppressed ω3 polyunsaturated fatty acids (PUFA) in membrane lipids and ω3 PUFA-derived anti-inflammatory oxylipins. Supplementing the WD with DHA lowered hepatic ARA in membrane lipids and ARA-derived oxylipins and significantly increased hepatic DHA and its metabolites in membrane lipids, as well as C_20–22_ ω3 PUFA-derived oxylipins. NASH markers of inflammation and fibrosis were inversely associated with hepatic C_20–22_ ω3 PUFA-derived Cyp2C- and Cyp2J-generated anti-inflammatory oxylipins (false discovery rate adjusted *p*-value; *q* ≤ 0.026). Our findings suggest that dietary DHA promoted partial remission of WD-induced NASH, at least in part, by lowering hepatic pro-inflammatory oxylipins derived from ARA and increasing hepatic anti-inflammatory oxylipins derived from C_20–22_ ω3 PUFA.

## 1. Introduction

Nonalcoholic fatty liver disease (NAFLD) is the most common chronic fatty liver disease worldwide [1,2,3] and is defined as excessive neutral lipid deposition in the liver in individuals who consume little or no alcohol [4,5]. Obesity and type 2 diabetes mellitus (T2DM) are strongly associated with NAFLD [3,6,7,8]. In fact, 60% of patients with a BMI > 30 display evidence of liver steatosis [9]. Based on estimates from the Centers for Disease Control, ~93 million adults [10] and ~14 million children [11] in the US are obese. As such, both obese children and adults are at risk of developing NAFLD [12]. Lifestyle, diet, genetics, and endocrine status contribute to the onset of NAFLD and its progression to nonalcoholic steatohepatitis (NASH), cirrhosis, and primary hepatocellular cancer (HCC) [7,13]. Moreover, NAFLD is a risk factor for cardiovascular disease [14,15,16]. The top four risk factors for NAFLD are obesity, T2DM, dyslipidemia, and metabolic syndrome [17,18].

The progression of benign steatosis to NASH is a multicellular and multi-hit process [19,20,21,22,23] that is associated with excessive lipid accumulation in hepatocytes leading to insulin resistance and hepatic injury involving endoplasmic reticulum stress, oxidative stress, and inflammation [24]. Hepatic injury leads to cell death and fibrosis [25,26,27]. The best strategies to prevent NAFLD and stop its progression from benign steatosis to NASH remain ill-defined [28,29,30,31]. While current strategies focus on lifestyle management (exercise and diet) [28,32,33,34,35,36,37,38,39,40,41,42,43], patient noncompliance remains a major concern when using lifestyle interventions to improve health outcomes [44,45,46]. Although targeted pharmacological agents are in development to treat NAFLD [46,47,48], adverse drug effects arising from off-target mechanisms often occur. To date, the Food and Drug Administration has not approved any specific therapies for NASH [49]. The absence of specific treatment strategies makes NAFLD a major public health concern [30].

While the clinical features of NAFLD are well described, the impact of diet, such as the western diet, on hepatic physiology and lipid metabolism remains poorly defined. Accordingly, we developed a preclinical NASH model using the low density lipoprotein (LDL)-receptor null (*Ldlr ^-/-^*) mouse and the western diet (WD). This model recapitulates human NASH in male and female mice [50,51,52]. Mice fed the WD become obese and the liver presents all the hallmarks of NASH, i.e., hepatosteatosis, leukocyte accumulation in the liver, centrilobular fibrosis, and increased expression of HCC markers.

A key outcome of our research established that the WD lowers hepatic content of C_18-22_ polyunsaturated fatty acids (PUFA, both ω3 and ω6). The WD is moderately high in saturated (SFA) and monounsaturated (MUFA) fatty acids, simple sugar and cholesterol, but low in essential fatty acids, e.g., linoleic acid (LA, 18:2,ω6) and α-linolenic acid (ALA, 18:3,ω3) [50,53,54]. Interestingly, clinical studies have shown that NASH patients have low hepatic C_18-22_ PUFA when compared to patients with benign steatosis [55,56,57]. Moreover, PUFA and ω3 PUFA, specifically, affect whole body lipid metabolism by decreasing blood triglycerides, suppressing fatty acid synthesis, and promoting fatty acid oxidation. In contrast, dietary ω6 PUFAs are precursors to bioactive pro-inflammatory oxylipins [58,59]. As such, changes in the relative abundance of hepatic SFA, MUFA, PUFA, and the type of PUFA, i.e., ω3 versus ω6 PUFA, has the potential to affect whole body and liver health.

To reinforce the role of dietary PUFA in NAFLD development, we established that supplementing the WD with docosahexaenoic acid (DHA, 22:6,ω3) at 2% total calories restored hepatic C_20–22_ ω3 PUFA, lowered arachidonic acid (ARA, 20:4,ω6), a precursor to harmful pro-inflammatory ARA-derived oxylipins, and lowered histologic and transcriptomic markers of inflammation, oxidative stress, and fibrosis [52]. More recently, we used a lipidomic approach to assess the impact of the WD on hepatic membrane lipids and oxylipins in female *Ldlr ^-/-^* mice [60]. These studies established that feeding mice the WD significantly changed the acyl chain composition of multiple hepatic membrane lipid classes and ω3 and ω6 PUFA-derived oxylipins. Specifically, the WD increased the hepatic membrane content of SFA and MUFA, as well as ARA and ARA-derived oxylipins. The hepatic abundance of ω3 PUFA-derived oxylipins, however, was low in mice fed the WD. This oxylipin profile was associated with increased hepatic markers of inflammation, oxidative stress, fibrosis, apoptosis, autophagy, notch and hedgehog signaling, and hepatic cancer [60]. 

Fatty acids and their derivatives are well-established regulators of cell function. The principal targets for this action include regulation of membrane lipid composition, oxylipin type and abundance, and regulation of cell signaling originating from the plasma membrane, as well as targeting nuclear receptors [59,61]. The rationale for using DHA to combat NASH is based on the well-established role of C_20–22_ ω3 PUFA in the control of blood triglycerides and hepatic fatty acid synthesis and oxidation [62]. Moreover, C_20–22_ ω3 PUFA interfere with ARA-derived oxylipin production and function [58]. Finally, clinical studies support the use of ω3 PUFA dietary supplementation to treat NAFLD [12,47]. Recent meta-analyses of clinical trials using C_20–22_ ω3 PUFA dietary supplementation indicate significant improvement in several metabolic outcomes, including lowering plasma triglycerides and hepatic fat content [12,47]. The most consistent improvement in liver health is seen with dietary DHA [12] or the combination of DHA and eicosapentaenoic acid (EPA, 20:5, ω3), e.g., Lovaza^TM^, GlaxoSmithKline [63]. EPA treatment alone, however, has proven ineffective in improving liver health in NAFLD patients [50,64]. 

**Figure 1:**
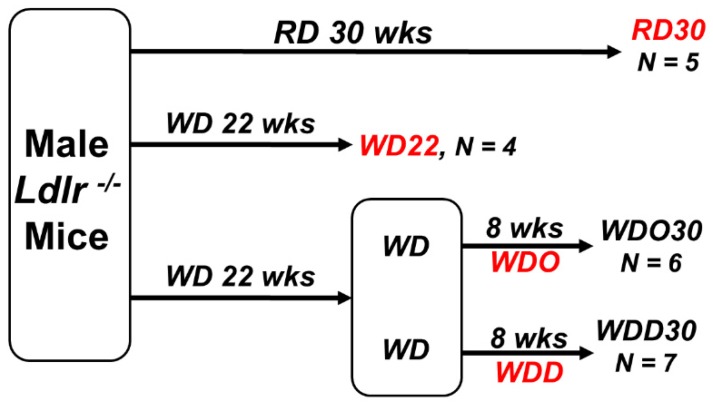
Study design for docosahexaenoic acid (DHA)-mediated nonalcoholic steatohepatitis (NASH) remission in male *Ldlr ^-/-^* mice. Liver samples used in this lipidomic analysis were obtained from our previously published study assessing the capacity of DHA to promote NASH remission [52]. Briefly, mice at 10 wks of age were fed a chow diet (Purina Pico Lab diet 5053) and served as a reference diet (RD) group. The RD group was maintained on the RD for the duration of the study, i.e., 30 weeks (wks; RD30, number of animals (*N*) = 5). Mice were also fed the western diet (WD) (Research Diets, D12079B) for 22 wks. At 22 wks, a group of WD-fed mice were euthanized for recovery of blood and liver. This group (WD22, *N* = 5) served as a baseline for disease progression. The remaining WD-fed mice were switched to a WD supplemented with either olive oil (WDO30, *N* = 6) or DHASCO (WDD30, *N* = 7) and euthanized 8 weeks (8 wks) later. See Materials and Methods for more details.

In this report, we used liver samples from our previous study which documented the capacity of DHA to block NASH progression (Figure 1) [52]. This study included detailed gas chromatographic (GC) analysis of diet effects on hepatic lipids as well as extensive transcriptomic analysis of hepatic markers of inflammation and fibrosis. Herein, we expanded our lipidomic analysis by using ultra-high-performance liquid chromatography coupled with tandem mass spectrometry (UPLC-MS/MS) and high-performance liquid chromatography coupled with dynamic multi-reaction monitoring (HPLC-dMRM), as described [60]. Our aim was to document how the WD and DHA altered hepatic membrane lipid and non-esterified oxylipin composition in a preclinical NASH model. We then used a statistical approach to determine how these diet-induced changes in hepatic lipids correlated with changes in hepatic markers of inflammation and fibrosis.

## 2. Results 

### 2.1. Impact of the Western Diet (WD) on Membrane Lipids

The study designed included 4 groups of male *Ldlr^-/-^* mice as described in Figure 1. A control group consisted of mice maintained on a reference diet (RD) for 30 weeks. The remaining mice were fed the WD for 22 weeks. At 22 weeks, WD-fed mice were split into three groups. One group was euthanized and served as the baseline group (WD22) for NASH progression analysis. The remaining mice were fed the WD supplemented with either olive oil (WDO) or DHASCO (WDD) for 8 weeks (see Materials and Methods). The WDO and WDD diets were matched for calories as fat. The dose of DHA used in these studies was equivalent to a human taking 4 g of Lovaza^™^ (GlaxoSmithKline)/day to treat hypertriglyceridemia [65]. After 8 weeks on the WDO and WDD diets, mice were euthanized for blood and liver collection. The group identifications for these mice are WDO30 and WDD30, respectively. Total hepatic lipids were extracted and fractionated using the UPLC-MS/MS approach described in Materials and Methods. 

**Figure 2:**
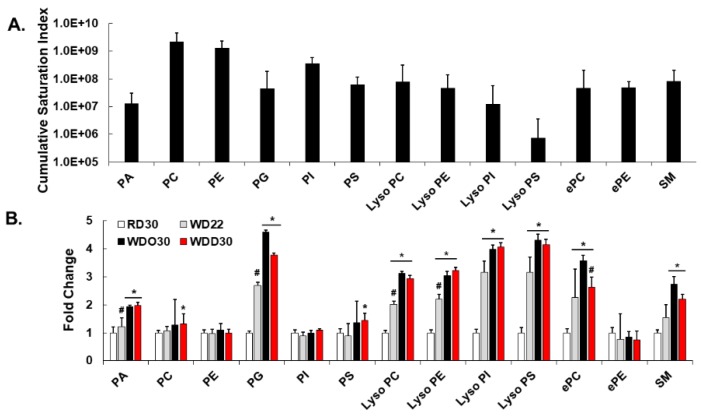
Diet effects on membrane lipids. (**A**): Cumulative saturation index of lipids in each lipid class. The saturation index (SI) was calculated as follows: one minus (number of double bonds) divided by (number of fatty acyl carbons minus one). The cumulative saturation index was calculated by multiplying the SI by the peak intensity of each lipid species and summing all lipids within each lipid class. (**B)**: Effect of diet on the cumulative saturation index for each lipid class. Results are presented as fold change, mean ± standard error of the mean (SEM); *, *q* < 0.05 vs. RD30; #, *q* < 0.05 vs. WDO30; [*q*-value is the false discovery rate (FDR) adjusted *p*-value].

Our UPLC-MS/MS analysis identified 13 classes of membrane lipids (Figure 2). To assess the impact of diet on these lipids, we quantified the cumulative saturation index (CSI). The CSI reflects the amount of lipid within a lipid class and the level of saturation of the fatty acyl chains within each lipid class. Figure 2A represents the CSI across all major membrane lipids in mice maintained on the RD for 30 weeks (RD30). The highest CSI was in the lipid classes including phosphatidyl choline (PC) and phosphatidyl ethanolamine (PE) and the lowest in lysophosphatidyl serine (lyso PS). 

The impact of the WD and DHA on CSI is shown in Figure 2B. Since the WD is enriched in SFA and MUFA, we expected a significant increase in the CSI. Accordingly, feeding mice the WD increased the CSI in phosphatidic acid (PA), phosphatidyl glycerol (PG), phosphatidyl serine (PS), all lysophospholipids [(lysophosphatidyl choline (lyso PC), lysophosphatidyl ethanolamine PE (lyso PE), phosphatidyl inositol (lyso PI), phosphatidyl serine (lyso PS)], ether phosphatidyl choline (ePC), and sphingomyelin (SM), but not in PC, PE, phosphatidyl inositol (PI), or ether phosphatidyl ethanolamine (ePE). PA is a precursor to multiple membrane lipids, while PG is a precursor to cardiolipins. Surprisingly, including DHA in the WD significantly lowered the CSI (by 20%) in only one lipid class, ePC. Yet, a detailed examination of all lipid species within each lipid class revealed assimilation of DHA and its metabolites (20:5, ω3; 22:5, ω3) in all lipid classes, except SM (Table S1: diet effects on all lipids). While the WD significantly increased the CSI as a result of increased dietary SFA and MUFA content, DHA and its metabolites had little impact on the overall CSI of most lipid classes.

**Figure 3:**
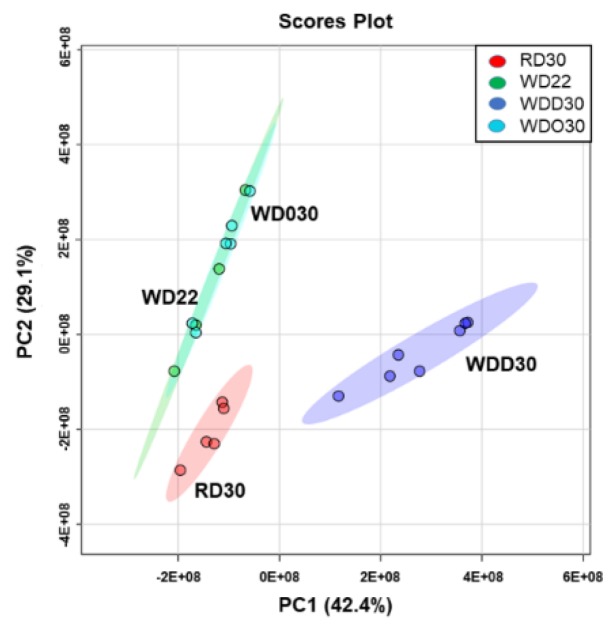
Principal component analysis of diet effects on hepatic lipids. All lipid data collected for this study and our previous study [52] was used in this analysis. The data included fatty acid methyl esters reported previously [52] and all membrane lipid and oxylipin data obtained by UPLC-MS/MS and HPLC-dMRM analysis, respectively. The principal component analysis was carried out using the statistical package in Metabolanalyst 4.0 [66].

We next used a statistical approach to establish differences between treatment groups. Accordingly, all lipid data from the UPLC-MS/MS and HPLC-dMRM analysis plus our previous GC analysis [52] was subject to a principal component analysis (PCA) (Figure 3). While four groups were included in our study, the PCA revealed only three clusters. Two clusters (WD22 and WDO30) overlapped indicating that these groups differed little in terms of lipid composition. The lipid composition of the WD22 and WDO30 groups clearly differed from the reference diet group (RD30). Interestingly, the WDD30 group does not overlap with either the RD30 or WD22 and WD30 clusters, reflecting its unique lipid composition. This outcome indicates that 8 wks of DHA treatment does not restore hepatic membrane lipid acyl chain composition to that seen in the RD30 group. 

**Figure 4:**
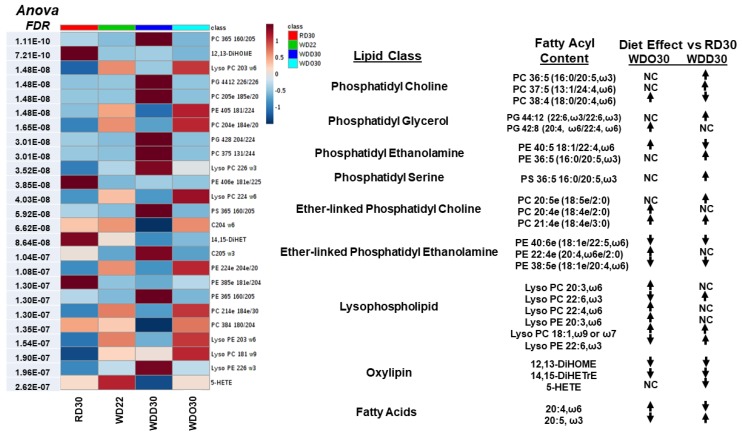
Heat map illustrating diet effects on the top 25 lipid features. As in Figure 3 all lipid data was used to perform an ANOVA (one-way) and a heat map was constructed using the statistical package in Metabolanalyst features [66]. The top 25 highly significant lipid features are illustrated in the heat map. The *q*-value for each lipid is on the left side of the heat map. Columns at the right list specific lipids within each lipid class that were on the heat map. Arrows indicate the effect of the diet WDO30 and WDD30 when compared to the RD30 group (increase, decrease, no change (NC)) (WD supplemented with either olive oil (WDO) or DHASCO (WDD)).

Further analysis identified the top 25 highly significant differences (*q* ≤ 3.0 × 10^−7^) in lipid composition amongst the four groups (Figure 4 and Table S2: Lipids significantly affected by diet). A key result of this analysis was that the WD increased 20:4, ω6 and its metabolites in multiple membrane lipids (PC 38:4, PG 42.8; PE 40:5, lyso PC 20:3; lyso PC 22:4; lyso PE 20:3), but lowered oxylipins derived from linoleic acid (18:2, ω6), i.e., 12,13-DiHOME. This finding replicates our previous results documenting the effects of the WD on hepatic lipids derived from WD-fed female *Ldlr ^-/-^* mice [60].

Addition of DHA to the WD not only increased the abundance of DHA and its metabolites in hepatic phospholipids (PC 36:5, PG 44:12, PS 36.5, lyso PC 22:6, lyso PE 22:6), but also decreased levels of specific membrane lipids containing 20:4,ω6 and its metabolites (PC 38:4, PE 40:5, PE 38.5). Such effects of dietary ω3 PUFA on membrane lipid composition are not new. What is new, however, is the impact of WD and DHA on a broad range of hepatic lipids, including phosphoglycerol lipids (PC, PE, PG, PI, PS), ether lipids (ePC, ePE), lysophospholipids (lyso-PC, -PE, -PI, -PS), and sphingolipids. The sphingolipids were the only lipid class significantly affected by WD and DHA, but found to contain no C_20–22_ PUFA (both ω3 and ω6) (Table S1: Diet effects on all lipids). As such, DHA mediated effects on SM acyl chain content likely involves DHA regulation of hepatic abundance of SFA and MUFA, as well as the incorporation of these fatty acyls into SM. 

### 2.2. Diet Effects on Hepatic Non-Esterified Oxylipins

**Figure 5:**
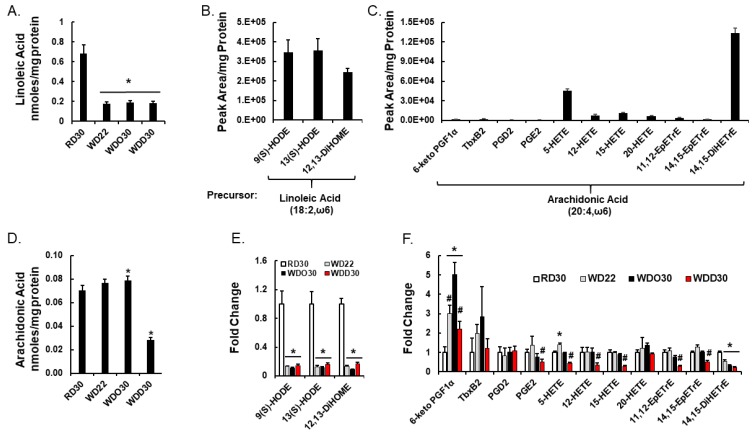
Diet effects on oxylipins derived from ω6 polyunsaturated fatty acids (PUFA). (**A**,**D**): Hepatic content of linoleic acid (LA; 18:2, ω6) and arachidonic acid (ARA; 20:4, ω6) as determined by gas chromatographic (GC) analysis. Results are presented as nmoles/mg protein, mean ± SEM. Oxylipins derived from LA (**B**) and ARA (**C**) were quantified, by HPLC-dMRM as described in the Materials and Methods Section. Liver samples were derived from the RD group and oxylipin levels are presented as the peak area/mg protein, mean ± SEM. Effects of diet on specific oxylipins are presented in (Panels E,F) as Fold Change, mean ± SEM. (Panels **E**,**F**) present LA- and ARA-derived oxylipins, respectively. *, *q* < 0.05 vs. RD30; #, *q* < 0.05 vs. WDO30. 9(S)-HODE, 9(S) hydroxyl octadecadienoic acid; 13(S)-HODE, 13(S) hydroxyl octadecadienoic acid; 12,13-DiHOME, 12,13-dihydroxy octadecenoic acid; 6-keto PGF1α, 6-keto prostaglandin F1α; TbxB2, thromboxane B2; PGD2, prostaglandin D2; PGE2, prostaglandin E2, 5-HETE, 5-hydroxyeicosatrienoic acid; 12-HETE, 12-hydroxyeicosatrienoic acid; 15-HETE, 15-hydroxyeicosatrienoic acid; 20-HETE, 20-hydroxyeicosatrienoic acid; 11,12-EpETrE, 11,12 epoxyeicosatrienoic acid; 14,15-EpETrE, 14,15 epoxyeicosatrienoic acid; 14,15-diHETrE, 14,15-dihydroxyeicosatrienoic acid.

**Figure 6:**
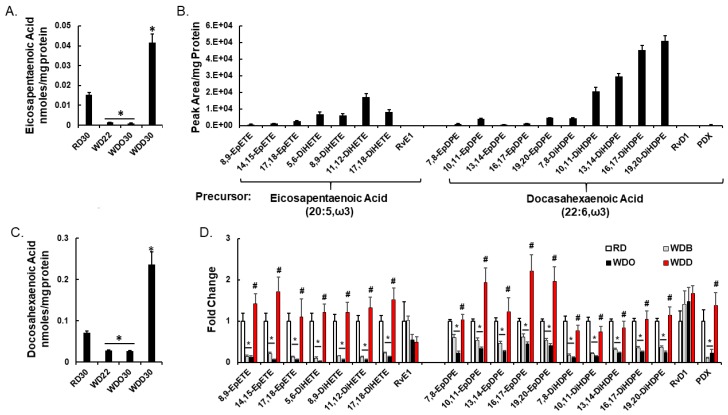
Diet effects on oxylipins derived from ω3 PUFA. Hepatic content of eicosapentaenoic acid (EPA; 20:5, ω3) (Panel **A**) and docosahexaenoic (DHA; 22:6, ω3) (Panel **C**). Hepatic EPA and DHA were quantified as described in the Materials and Methods Section and presented as the mean ± SEM of nmoles/mg hepatic protein. (Panels **B**,**D**): Effects of diet on EPA and DHA derived oxylipins, respectively. Results are presented as Peak area/mg protein (Panel **B**) and Fold Change, mean ± SEM (Panel **D**). *, *q* < 0.05 vs. RD30; #, *q* < 0.05 vs. WDO30. 8,9-EpETE, 8,9-epoxyeicosatetraenoic acid; 14,15-EpETE, 14,15-epoxyeicosatetraenoic acid; 17,18-EpETE, 17,18-epoxyeicosatetraenoic acid; 5,6-DiHETE, 5,6-dihydroxyeicosatetraenoic acid; 8,9-DiHETE, 8,9-dihydroxyeicosatetraenoic acid; 11,12-DiHETE, 11,12-dihydroxyeicosatetraenoic acid; 17,18-DiHETE, 17,18-dihydroxyeicosatetraenoic acid; RvE1, resolvin E1; 7,8-EpDPE, 7,8-epoxydocosapentaenoic acid; 10,11-EpDPE, 10,11-epoxydocosapentaenoic acid; 13,14-EpDPE, 13,14-epoxydocosapentaenoic acid; 16,17-EpDPE, 16,17-epoxydocosapentaenoic acid; 19,20-EpDPE, 19,20-epoxydocosapentaenoic acid; 7,8-DiHDPE, 7,8-dihydroxydocosapentaenoic acid; 10,11-DiHDPE, 10,11-dihydroxydocosapentaenoic acid; 13,14-DiHDPE, 13,14-dihydroxydocosapentaenoic acid; 16,17-DiHDPE, 16,17-dihydroxydocosapentaenoic acid; 19,20-DiHDPE, 19,20-dihydroxydocosapentaenoic acid; RvD1, resolvin D1; PDX, protectin DX.

**Figure 7:**
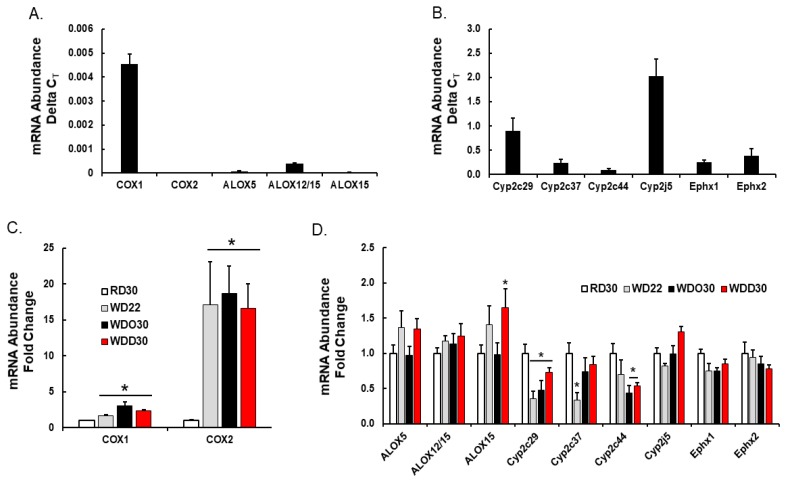
Diet effects on hepatic enzymes involved in oxylipin metabolism. Hepatic RNA was extracted, converted to cDNA and used to quantify transcript abundance using qRTPCR as previously described [52]. The primers used to measure each transcript were previously described [51,60]. Cyclophilin was used as the reference gene. (Top panels **A**,**B**): Relative abundance of transcripts encoding enzymes involved in hepatic oxylipin metabolism. Results are presented as delta C_T_, mean ± SEM. (Lower panels **C**,**D**): Diet effects on hepatic transcripts encoding enzymes involved in oxylipin metabolism. Results are presented as Fold Change, mean ± SEM; *, *q* < 0.05 vs. RD30. COX, cyclooxygenase; ALOX, arachidonic lipoxygenase; CYP, cytochrome P450, Ephx1, microsomal epoxide hydrolase; Ephx2, soluble epoxide hydrolase.

**Figure 8:**
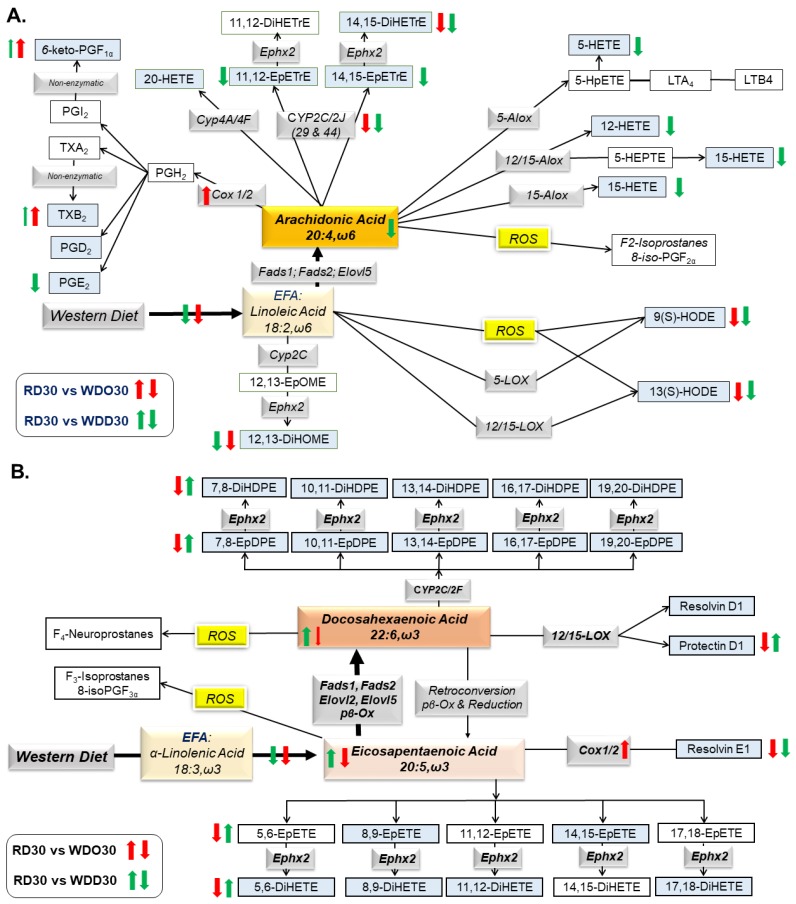
Summary of WD and DHA effects on hepatic oxylipins derived from ω6 PUFA (**A**) and ω3 PUFA (**B**). The diagrams illustrate the pathway for the conversion of dietary essential fatty acids to C_18-22_ PUFA and the conversion of PUFA to oxylipins. The pathways are modified from pathways published by Gabbs et al. [61]. Oxylipins highlighted in blue represent oxylipins that were quantified by LC/MS or gas chromatography (Figure 5 and Figure 6). Enzymes involved in oxylipin metabolism are in gray boxes (Figure 7). Red and green arrows are used to represent the effects (increase; decrease) of the WDO versus RD30 (red arrows) and WDD30 versus RD30 (green arrows) on hepatic abundance of fatty acids, oxylipins, and transcripts involved in PUFA and oxylipins metabolism. Thin and thick arrows represent a weak and strong response to diet, respectively. EFA: essential fatty acids; ROS, reactive oxygen species. *Fads*, fatty acid desaturase; *Elovl*, fatty acid elongase; pβOx, peroxisomal β-oxidation; *COX*, cyclooxygenase; *ALOX*, arachidonic lipoxygenase; CYP, cytochrome P450, *Ephx1*, microsomal epoxide hydrolase; *Ephx2*, soluble epoxide hydrolase.

Intrahepatic non-esterified oxylipins arise from phospholipase-mediated excision of fatty acyls from membrane lipids. These non-esterified fatty acids serve as substrates for cell-specific pathways generating oxylipins that, in turn, serve as regulatory ligands for G-protein receptors (GPR) and nuclear receptors [61,66]. Herein, we examined the effect of the WD and DHA on hepatic oxylipins derived from LA, ARA, EPA, and DHA (Figure 5 and Figure 6) and the expression of enzymes involved in oxylipin metabolism (Figure 7); results are summarized in Figure 8. 

We previously reported that feeding female *Ldlr ^-/-^* mice fed the WD decreased hepatic oxylipins derived from linoleic acid (LA, 18:2, ω6), but increased hepatic oxylipins derived from arachidonic acid (ARA, 20:4, ω6) [60]. As illustrated in Figure 5, the WD and WDO diets had similar effects on hepatic oxylipins in male *Ldlr ^-/-^* mice as seen in female mice [60]. Our oxylipin analysis identified 14 ω6 PUFA-derived oxylipins; three from LA and 11 from ARA (Figure 5). Three oxylipins, 12,13-DiHOME; 14,15-DiHETE; and 5-HETE ranked in the top 25 highly significantly lipids affected by the WD (Figure 4). The dihydroxy fatty acid, 12,13-DiHOME, is one of three LA-derived oxylipins, and 14,15-DiHETE is one of eleven ARA-derived oxylipins identified in our analysis (Figure 5 and Figure 8). These dihydroxy oxylipins are generated by the action of a soluble epoxide hydrolase (Ephx2) action on epoxy fatty acids, i.e., 12,13-EpHOME and 14,15-EpETrE, respectively. The LA and ARA derived epoxides are generated by hepatic epoxygenases (Cyp2C; Cyp2J). Highly abundant ω6 PUFA-derived oxylipins include 9(S)-HODE, 13(S)HODE, 12,13-DiHOME, 5-HETE, and 12,15-DiHETrE, while low abundance oxylipins include 6-keto PGF1α, TBXB2, PGD2, PGE2, and HETEs (12-, 15-, 20-HETE) and 14,15-EpETrE. WD or WDO feeding increased hepatic ARA and significantly increased 6-keto PGF1α and TBX2B, but had little effect on other 20:4, ω6 derived oxylipins (Figure 5C,D).

We identified eight and 12 oxylipins derived from EPA and DHA, respectively, in male mice fed the RD, i.e., RD30 group (Figure 6 and Figure 8). Feeding female *Ldlr^-/-^* mice the WD lowered hepatic levels of all ω3 PUFA-derived oxylipins [60]. Male *Ldlr ^-/-^* mice fed the WD or WDO resulted in significantly lower levels of all hepatic ω3-PUFA-derived oxylipins (Figure 6). The decline in these oxylipins paralleled the WD-mediated decline in hepatic EPA and DHA (Figure 6A,C). 

Supplementing the WD with DHA, i.e., WDD30 group, had no effect on hepatic LA or LA-derived oxylipin abundance. The WDD, however, significantly lowered hepatic ARA and all ARA-derived oxylipins, except PGD2 and 20-HETE (Figure 5D,F). The decline in hepatic 20:4, ω6-derived oxylipins paralleled the DHA-mediated suppression of hepatic 20:4, ω6 content (Figure 5D). Clearly, the WD has a potent effect on hepatic oxylipin type and abundance. Supplementing the WD with DHA had an equally potent effect on hepatic ω3 PUFA-derived oxylipins by reversing the WD effect on ARA-derived and C_20–22_ ω3 PUFA-derived oxylipins.

We next examined the diet effects on hepatic enzymes involved in generating hepatic oxylipins (Figure 7). Cyclooxygenases (Cox1, Cox2) and arachidonic acid lipoxygenases (Alox5, Alox12/15, Alox15) are expressed at low levels in mouse liver (Figure 7A), whereas enzymes generating fatty epoxides (Cyp2C29, Cyp2C37, Cyp2C44, Cyp2J5) and dihydroxy fatty acids (Ephx1, Ephx2) are highly expressed in liver (Figure 7B). The differential expression of these enzymes likely reflects cell specific expression in the liver. For example, Cox1 and Cox2 are expressed in liver, but not in hepatic parenchymal cells, i.e., hepatocytes. These enzymes are likely expressed in resident macrophage (Kupffer cells) and infiltrating leukocytes. Hepatocytes, however, express receptors for Cox products, e.g., EP4, and respond to changes in oxylipins through paracrine mechanisms [67].

Feeding mice the WD and WDO resulted in the induction of both Cox1 and Cox2, but the WD and WDO diets had no significant effects on the Alox subtypes (5-, 12/15-, 15-Alox) (Figure 7). Supplementing the WD with DHA did not attenuate Cox1 or Cox 2 expression. If Cox 1 and Cox 2 expression parallels Cox 1 and 2 activity, then the decline in Cox-products, e.g., prostacyclin (PGI2, precursor of 6-keto PGF1α), PGE2, and thromboxane A2 (precursor of TBXB2) cannot be explained by a suppression of enzyme expression. As such, our data suggest that the DHA-mediated decline in Cox products may be due, at least in part, to the DHA-mediated suppression of hepatic ARA levels, particularly in membrane lipids (Figure 4 and Figure 5). Cyp2c29, Cyp2c37 and Cyp2c44 expression was suppressed ~50% by the WD. Only Cyp2c29 expression was partially restored by the addition of DHA to the diet. Neither WDO nor WDD affected the expression of Cyp2J or Ephx subtypes.

### 2.3. Associations between Hepatic Lipids and NASH Markers of Inflammation and Fibrosis

We previously reported that the WD promoted hepatic inflammation and fibrosis, while addition of DHA to the WD blocked disease progression by attenuating expression of inflammation and fibrosis markers [52]. Herein, we asked if changes in specific transcriptomic markers of inflammation and fibrosis were associated with changes in membrane lipids and oxylipins. Accordingly, we used an unbiased statistical approach, i.e., Pattern Hunter in Metabolanalyst 4.0 [66], to identify associations between diet-induced changes in membrane lipids, oxylipins, and NASH pathology, i.e., inflammation and fibrosis (Table 1 and Table 2). The transcriptomic data for this analysis was from our previous study [52], the same study used for the current lipidomic analysis. Results are presented as the top 10 positive and negative associations between specific lipids and markers of inflammation (Table 1: osteopontin (*Opn*), monocyte chemoattractant protein 1 (*Mcp1*), cell differentiation 68 (*CD68*)) and fibrosis (Table 2: collagen 1A1 (*Col1A2*), tissue inhibitor metalloprotease 1 (Timp1) and lysyl oxidase (Lox)). 

#### 2.3.1. Inflammation

Lipids positively associated with *Opn* expression include phosphatidyl glycerol (PG) containing MUFA and PUFA (ω6 > ω3) while 50% of the lipids negatively associated with *Opn* expression were ω3 PUFA-derived oxylipins (Table 1). In contrast, lipids positively associated with *Mcp1* and *CD68* include no PG, but several ether lipids (e.g., PC 16:0e; PC 18:1e) containing predominantly MUFA and short and long chain SFAs. Lipids negatively associated with *Mcp1* and *CD68* expression include oxylipins (12,13-DiHOME, 14,15-DiHETrE) and membrane phospholipids (PA, PE, PI) containing C_18–22_ ω3 and ω6 PUFA. These association studies indicate that elevated expression of inflammation markers was associated with increased membrane abundance of C_18–20_ MUFA and C_20–22_ ω6 PUFA, while attenuated expression of these markers was associated with increased membrane content of C_18–22_ ω3 and ω6 PUFA and hepatic levels of ω3 and ω6 PUFA derived oxylipins. The epoxygenase and epoxide hydrolase pathways (Cyp2C, Cyp2J and Ephx2) rather than the Cox/Alox and oxidative stress pathways generate the majority of these oxylipins. In addition, lipids associated (positively and negatively) with *Opn* expression are clearly distinct from the lipids associated with *Mcp1* and *CD68* expression, suggesting different membrane-associated mechanisms involved in the expression of these inflammation markers.

#### 2.3.2. Fibrosis

Lipids positively and negatively associated with the expression of *Col1A2* and *Timp1* are nearly identical; and include membrane lipids (lyso PA, lyso PE, lyso PC, SM) containing C_14–16_ SFA, C_18_ MUFA, and C_18–22_ ω6 PUFA, but no ω3 PUFA. Lipids negatively associated with *Col1A2* and *Timp1* expression include DHA derived oxylipins (7, 8-DiHDPE; 10, 11-DiHDPE) and membrane lipids (PA, PC, PE, PI) containing C_16–22_ MUFA and C_18–20_ ω6 PUFA. Interestingly, lipids positively and negatively associated with Lox expression are remarkably similar to those associated with *Opn* expression. PG containing MUFA and C_20–22_ ω6 PUFA are positively associated with *Lox* expression, while 60% of the lipids negatively associated with Lox expression are oxylipins derived from ARA and EPA. This outcome may reflect common membrane-associated mechanisms associated with *Lox* and *Opn* expression.

## 3. Discussion

The aim of this study was to use a lipidomic approach to identify potential lipid mediators of inflammation and fibrosis associated with WD-induced NASH and DHA-mediated NASH remission. Accordingly, we identified and quantified hepatic membrane lipids and non-esterified oxylipins in a preclinical mouse model of NASH. Feeding mice the WD significantly increased the saturation index of many, but not all membrane lipids (Figure 2). The WD increased SFA and MUFA in several membrane lipids, i.e., PA, PG, lyso PC, lyso PE, lyso PI, lyso PS, ePC, and SM. Surprisingly, addition of DHA to the WD had little effect on the membrane saturation index, despite the fact that DHA and its metabolites were assimilated into all lipid classes, except SM (Table S1). We suspect the assimilation of DHA and its metabolites into membranes has local effects on membrane fluidity, lipid raft composition, and membrane cholesterol content that potentially affects receptor-mediated mechanisms emanating from membranes [58,59].

On a more granular level, we identified strong associations between diet-induced changes in hepatic membrane lipid composition, membrane derived signaling molecules, and the expression of genes linked to WD-induced hepatic inflammation and fibrosis (Table 1 and Table 2). We identified two membrane derived lipid classes that are known to play a role in cell signaling, i.e., lysophospholipids (Lyso PL) and oxylipins. Lyso PLs form in the process in *de novo* membrane lipid synthesis (Kennedy Pathway) and membrane lipid remodeling (Lands Pathway). Our untargeted lipidomic analysis cannot distinguish between these pathways, nor can it distinguish between acyl chains in the sn1 or sn2 positions of the lyso PLs. Dietary fat content clearly affected the acyl chain composition of these lyso PLs (Figure 4). Oxylipin precursors, i.e., non-esterified fatty acids (NEFA), are generated as a result of membrane remodeling and involves phospholipase activation. NEFA excised from membranes are substrates for several enzymatic pathways that are active in the liver, including cyclooxygenases (Cox (-1 and -2), arachidonate lipoxygenases (Alox (-5, -12/15, -15)), cytochrome P450 class 2 ((Cyp2 (C and J)), and epoxide hydrolases (Ephx 1 and 2) (Figure 8). 

Lyso PLs and oxylipins functioning as ligands have the potential to regulated cell function through multiple receptor-mediated mechanisms. Both bioactive lipids regulate cell function through G-protein receptors (GPR) and nuclear receptors. Lyso PLs signal through GPR23, GPR34, GPR44, GPR92, GPR93, and GPR174, while oxylipins signal through GPR for eicosanoids (prostaglandins (PGE2), thromboxanes (TBXA2), and prostacyclins (PGI2)). Lyso PLs and oxylipins also signal through nuclear receptors, e.g., PPARα, β/δ, γ1, γ2) [68,69,70,71,72,73,74,75]. Our studies clearly establish that both the WD and DHA have major effects on membrane lipid composition and the type and abundance of hepatic lyso PLs and oxylipins derived from PUFA (Figure 4, Figure 5, Figure 6, Figure 7 and Figure 8). 

We took advantage of our transcriptomic data [52] to identify associations between membrane lipids, oxylipins, and markers of inflammation and fibrosis, key markers of NASH (Table 1 and Table 2). Hepatic levels of lyso PC and lyso PE containing C_20–22_ ω6 PUFA were positively associated with the expression of inflammation (Opn, CD68) and fibrosis (Col1A2, Timp1, LOX) markers (Table 1 and Table 2). As such, changes in the hepatic abundance of lyso PL enriched in C_20–22_ ω6 PUFA, acting through membrane GPR and/or nuclear receptors, may contribute to hepatic pathology. Key enzymes involved in lyso PL metabolism include phospholipases (PLA, multiple subtypes) and lysophosphatidyl choline acyl transferases (LpCAT; 4 subtypes). We previously established that hepatic phospholipase (PLA2g6) and LpCAT1 and LpCAT2 were induced by the WD, while DHA, but not EPA, suppressed LpCAT 1 and 2 expression [50]. Thus, DHA has the potential to regulate cellular levels of LpCAT-derived ligands controlling specific G-protein (GPRs) and nuclear receptors. 

Oxylipins represent the second group of regulatory lipids examined in this study (Figure 5, Figure 6, Figure 7 and Figure 8). Prostaglandins and leukotrienes are well-studied oxidation products of PUFA that are generated by cyclooxygenases (Cox) and lipoxygenases (Alox), respectively. The products of these enzymes are short-lived oxidized lipids that bind to and activated G-protein receptors (GPRs) that induce changes in intracellular second messengers, i.e., cAMP and calcium, affecting multiple signaling pathways [76]. These active products are rapidly degraded to relatively inactive compounds. Because of the short-lived nature of these active products, the inactive compounds are quantified as surrogates for in vivo synthesis of the bioactive Cox/Alox products [76]. Two 20:4, ω6-derived oxylipins identified in our analysis include 6-keto-PGF1α and TXB2; these are degradation products of PGI2 and TXA2, respectively. PGI2 and TXA2 are involved in platelet aggregation, vasodilation, and inflammation, while PGD2 and PGE2 are involved in inflammation and vasodilation [61]. Increased hepatic levels of these products are associated with the induction of expression of Cox1 and Cox2 mRNAs in response to the WD (Figure 7). Dietary DHA, however, lowers hepatic TBX2, 6-keto-PGF1α, and PGE2. This response parallels DHA-mediated suppression of hepatic ARA levels, as opposed to DHA-mediated suppression of Cox1 and Cox2 expression (Figure 5 and Figure 7). Alox products (5 HETE, 12-HETE and 15-HETE) are also lower in livers of WDD-fed mice. Like the Cox products, hepatic levels of Alox products paralleled changes in hepatic ARA. 

Other ω6 PUFA derived oxylipins include 9(S)-HODE and 13(S)-HODE, both of which are derived from linoleic acid by enzymatic (Alox) and non-enzymatic (oxidative stress) pathways. These products affect ER-stress, apoptosis, inflammation, cellular adhesions, and PPARγ function [77]. Since there was little effect of diet on hepatic Alox5, Alox12/15, or Alox15 expression, hepatic oxidative stress likely accounts for the increased hepatic levels of 9(S)-HODE and 13(S)-HODE in response to the WD [50]. The decline in these oxylipins parallel the WD-mediated suppression of hepatic LA content (Figure 5A). Feldstein et al. recently reported increased levels of these oxylipins in NASH patients, when compared to patients with benign steatosis [78]. This finding contrast with our findings and may reflect differences in hepatic oxidative stress management in human versus mouse livers. This group also reported no significant change in 5-HETE, 12-HETE, or 15-HETE in normal, steatotic, or NASH livers of patients. These results are similar to our findings (Figure 5F). 

The other class of oxylipins examined included products generated by epoxygenases (Cyp2C, Cyp2J) and a soluble epoxide hydrolase (Ephx2). Like the Cox products, the epoxide products of Cyp2C and CYP2J are bioactive compounds. Moreover, Cyp2C and Cyp2J products are rapidly degraded to dihydroxy fatty acids with low bioactivity [79]. The WD has little effect on the formation of the ARA-derived products, but DHA lowered hepatic levels of the epoxy (11,12-EpETrE; 14,15-EpETrE) and dihydroxy (14,15-DiHETrE) products derived from ARA. Since there was little effect of diet on the expression of the Cyp2C, Cyp2J, and Ephx2 (Figure 7), we attribute the declined in Cyp2C, Cyp2J, and Ephx2 products to DHA-mediated suppression of hepatic ARA content (Figure 5). However, we cannot exclude post-translational mechanisms controlling the activity of these enzymes. 

ARA-derived epoxygenase products are anti-inflammatory, pro-resolving bioactive mediators [79]. A decline in the hepatic abundance of these metabolites suggest an increase in hepatic inflammation. However, we previously reported that the WD increased hepatic markers of inflammation while the WD supplemented with DHA suppressed hepatic inflammation [52]. To explain this outcome, our analysis revealed a massive suppression (> 70%) of C_20–22_ ω3 PUFA-derived oxylipins (Figure 7 and Figure 8B) in livers of WDO30 fed mice. In WDD30 fed mice, however, the Cyp2C and Cyp2F-derived ω3-PUFA oxylipins were restored to levels at or above levels seen in mice fed the RD. Changes in C_20–22_ ω3 PUFA-derived oxylipins are inversely associated with transcriptomic markers of hepatic inflammation and fibrosis (*q* ≤ 0.026; Table 1 and Table 2). The concept of an inverse association between tissue levels of C_20–22_ ω3 PUFA and inflammation is not new [80]. In fact, other investigators using the choline-methionine-deficient rat [81] and mouse [82] models of NAFLD reported a similar inverse association between tissue levels of DHA and liver injury. Our studies extend these observations by showing how specific classes of bioactive lipids are responsive to diet and associated with NASH markers (Figure 4, Figure 5, Figure 6, Figure 7 and Figure 8; Table 1 and Table 2).

The outcome of our lipidomic analysis supports the notion that dietary supplementation with DHA mitigates WD-induced NASH progression, at least in part, by lowering hepatic pro-inflammatory oxylipins derived from C_20–22_ ω6 PUFA and increasing hepatic reparative/anti-inflammatory oxylipins derived from C_20–22_ ω3 PUFA. While there is limited information on the differential bioactivity of ω3 PUFA versus ω6 PUFA-derived Cyp2C and Cyp2J, Lopez-Vicario et al. reported that, when compared to ω6 PUFA-derived epoxides, ω3 PUFA-derived epoxides were more effective inhibitors of inflammation and autophagy in insulin sensitive tissues, like liver [83]. As such, tissue levels of ω3 PUFA-derived epoxides may be a good predictor of liver health status in the context of WD-induced NASH. Key next steps will be to identify mechanisms linking specific ω3 PUFA- and ω6 PUFA-derived oxylipins to the expression of specific genes involved in hepatic inflammation and fibrosis. 

## 4. Materials and Methods

### 4.1. Study Design for DHA-Mediated NASH Remission in Male Ldlr ^-/-^ Mice

This study was carried out in strict accordance with the recommendations in the Guide for the Care and Use of Laboratory Animals of the National Institutes of Health. All procedures for the use and care of animals for laboratory research were approved by the Institutional Animal Care and Use Committee at Oregon State University (Permit Number: A3229-01). Liver samples used in this lipidomic analysis were obtained from our previously published study assessing the capacity to DHA to promote NASH remission [52]. Briefly, male mice (B6:129S7-Ldlr^tm1Her/J^, stock# 002207 purchased from Jackson Labs) were group housed (4 mice/cage) and maintained on a 12 h light/dark cycle. Mice were acclimatized to the Oregon State University (OSU) animal facilities for 2 weeks before proceeding with experiments.

At 10 wks of age, mice were fed a chow (Purina Pico Lab diet 5053) and served as a reference diet (RD) group. The RD group was maintained of the RD for the duration of the study, i.e., 30 wks (RD30, *n* = 5) (Figure 1). *Ldlr ^-/-^* mice were also fed the western diet (Research Diets, D12079B). The WD consists of 41% energy as fat, 43% energy as carbohydrate, 17% energy as protein, and 0.15% w/w cholesterol [52]. After 22 wks on the WD, a group of WD-fed mice was euthanized for recovery of blood and liver. This group (WD22, *n* = 5) served as a baseline for disease progression. The remainder of the WD-fed mice were switched to a diet supplemented with olive oil or DHASCO. DHASCO is a dietary supplement provided by DSM Nutritional Products; it contains DHA in a triglyceride form. DHA represents ~40% of total acyl chains in DHASCO and DHASCO contains no EPA, DPA (22:5, ω3), ARA, or LA [50]. DHA is present in the diet at 2% total calories (WDD30, *n* = 7). In order to have isocaloric diets, olive oil was added to the WD diet, i.e., WDO30. The WDO30 and WDD30 groups were maintained on their respective diets for 8 wks. Mice were then fasted overnight and euthanized for the collection of liver and blood. All samples were stored at −80 °C until used for extraction. The design of this study allowed for the assessment of disease progression from 22 to 30 weeks and the capacity of DHA to affect disease progression (Figure 1). 

### 4.2. RNA Extraction and qRTPCR

Liver RNA was extracted using Trizol (Ambion by Life Technologies, Carlsbad, CA, USA), quantified, and used for qRTPCR as described previously [52]. Primers use for qRTPCR are described in our previous study [60]. Relative quantitation was determined using the delta C_T_ methods using cyclophilin as the reference gene. The delta C_T_ value was used for all statistical analyses.

### 4.3. Sample Preparation for Lipidomic Analysis

Liver lipids were extracted using a biphasic solvent system of cold methanol, methyl tert-butyl ether (MTBE), and water with some modifications [84]. Liver (~20–25 mg) was transferred to 2 mL pre-weighted polypropylene tubes containing ceramic beads and of LC–MS-grade cold methanol (240 μL). Deuterated lipid recovery standards (5 μL of Splash^®^ Lipidomix^®^ Mass Spec Standards (Avanti Polar Lipids, Alabaster, AL, USA) were added to each sample. Samples were homogenized in a Precellys^®^ 24 bead-based homogenizer for 2 min at 1350 rpm. Cold MTBE (750 μL) was added to the samples, followed by vortexing (10 s) and shaking (6 min) at 4 °C. Phase separation was induced by adding LC–MS-grade water (188 μL) followed vortexing and centrifugation (14,000 rpm, 2 min). The upper organic phase (300 μL) was recovered and evaporated using a Labconco centrivap vacuum concentrator (Kansas City, MO, USA). Dried lipid extracts were resuspended in a methanol/toluene (9:1, *v*/*v*, 100 μL) mixture containing CUDA (1-cyclohexyl ureido, 3-dodecanoic acid, 50 ng/mL; Cayman Chemical, Ann Arbor, MI, USA) as an additional internal standard. Samples were vortexed (10 s) and centrifuged (14,000 rpm, 2 min) prior to LC–MS/MS analysis.

### 4.4. Sample Preparation for Oxylipins Analysis

Oxylipins were extracted from liver using the approach described by Pedersen et al. [85], with minor modifications. Liver (~20–25 mg) was transferred 2 mL pre-weighted polypropylene tubes containing ceramic beads. Cold LC–MS-grade methanol (35 μL) and an anti-oxidant solution [0.2 mg mL^−1^ solution BHT (butylated hydroxytoluene) in 1:1 methanol:water] (5 μL) was added to each sample. Each sample also received 10 μL of a deuterated oxylipin recovery standard solution; the standards included 20 deuterated oxylipins (Table S1) in methanol at a concentration of 5 ng/µL each. Ten mM ammonium formate +1% formic acid in isopropanol (550 μL) and water (100 μL) was added and the tubes were placed in a Precellys^®^ 24 bead-based homogenizer for 2 min at 1350 rpm. Samples were centrifuged (9000 rpm for 5 min) at room temperature. Supernatants were transferred to a 96-well Ostro Pass Through Sample Preparation Plate (Waters Corp, Milford, MA, USA) and eluted into glass inserts containing 10 μL 20% glycerol in methanol by applying a vacuum (15 mm Hg) for 10 min. Eluents were dried by vacuum centrifugation in a Labconco centrivap vacuum concentrator for 2 h at room temperature. Once dry, samples were reconstituted with 100 μL of methanol: acetonitrile (50:50), containing the internal standard (CUDA at 50 ng/mL). Samples were transferred to a spin filter (0.22 μm PVDF membrane, Millipore-Sigma, Burlington, MA, USA) and centrifuged (3 min at 6 °C at 9000 rpm) before transferred to 2 mL amber LC–MS vials. Extracts were stored at −20 °C until analysis by ultra-performance liquid chromatography tandem mass spectrometry (UPLC–MS/MS). The internal oxylipin standards added to the samples (Table S3) were used to correct the recovery of the quantified oxylipins [86].

### 4.5. Chromatographic and Mass Spectrometry Conditions for Lipids and Oxylipins Analysis

#### 4.5.1. Untargeted Lipidomics

UHPLC was performed using a Shimadzu Nexera system (Shimadzu, Columbia, MD, USA) coupled to a triple time-of-flight (TOF)™ 5600 mass spectrometer (AB SCIEX, Framingham, MA, USA). Compounds were separated using a Waters Acquity UPLC CSH C18 column (100 mm length × 2.1 mm id; 1.7 μm particle size) with an additional Waters Acquity VanGuard CSH C18 pre-column (5 mm × 2.1 mm id; 1.7 μm particle size) held constant at 65 °C while utilizing a flow rate of 0.6 mL min^−1^. Resuspended samples were injected at 2 μL and 3 μL for electrospray ionization (ESI) positive and negative modes, respectively. To improve lipid coverage, different mobile phase modifiers were used for positive and negative mode analysis [87]. For positive mode, 10 mM ammonium formate + 0.1% formic acid was used, while 10 mM ammonium acetate (Sigma–Aldrich, St. Louis, MO, USA) was used for negative mode. Both positive and negative modes used the same mobile phase composition of (A) 60:40 *v*/*v* acetonitrile: water (LC–MS grade) and (B) 90:10 *v*/*v* isopropanol:acetonitrile. To enhance solubilization of ammonium formate and ammonium acetate after its addition in the mobile phase, the salts were dissolved first in small volume of water before their addition in the mobile phases (0.631 g ammonium formate or 0.771 g ammonium acetate/1 mL water/1 L mobile phase). Each mobile phase with modifiers was mixed, sonicated for 15 min to achieve complete dissolving of modifiers, mixed again, and then sonicated for another 15 min [88]. The separation was conducted under the following gradient: 0 min 15% (B), 0–2 min 30% (B), 2–2.5 min 48% (B), 2.5–11 min 82% (B), 11–11.5 min 99% (B), 11.5–12 min 99% (B), 12–12.1 min 15% (B), and 12.1–15 min 15% (B), at a flow rate of 0.6 mL min^−1^. All samples were kept at 4 °C throughout the analysis. 

All analyses were performed at the high-resolution mode in MS^1^ (~35,000 full width at half maximum (FWHM)) and at the high sensitivity mode (~15,000 FWHM) in MS^2^. Sequential window acquisition of all theoretical fragment-ion spectra (SWATH) in positive/negative ion mode was used as the data independent acquisition (DIA) system for all samples. Data dependent acquisition (DDA) on a separate quality control (QC) pool sample was used in order to verify the annotations from SWATH acquisition for the most abundant lipid species. Detailed information of SWATH conditions included in Supplemental Information entitled SWATH parameters for untargeted analysis.

The mass calibration was automatically performed every 6 injections using an APCI positive/negative calibration solution (AB SCIEX) via a calibration delivery system (CDS). Quality control was assured by (i) randomization of the sequence, (ii) injection of QC pool samples at the beginning and the end of the sequence and between each 10 actual samples, (iii) procedure blank analysis, and (iv) checking the peak shape and the intensity of spiked internal standards and the internal standard added prior to injection.

#### 4.5.2. Targeted Oxylipidomics

High Performance Liquid Chromatography (HPLC) was performed using a Shimadzu system (Shimadzu, Columbia, MD, USA) coupled to a QTRAP 4000 (AB SCIEX, Framingham, MA, USA). Employing dynamic multi-reaction monitoring (dMRM) we evaluated 39 oxylipins, 17 deuterated oxylipins, CUDA, and the deuterated surrogates eicosapentaenoic acid-d5 (EPA-d5), docosahexaenoic acid-d5 (DHA-d5), and arachidonic acid-d8 (ARA-d8) in a 22 min LC-run in a targeted approach (Figure S1). For each compound, optimal transitions were determined by flow injection of pure standards using the optimizer application, and transitions were compared to literature values when available for certain compounds. The detailed list of MRM transitions is in Table S4. In the dMRM acquisition mode the triple quadrupole MS system focuses directly on the expected analyte retention time (RT) with a defined detection window instead of user-defined time segments to capture groups of closely eluting compounds. Establishing a constant cycle time for each transition improves peak symmetry and allows for a more accurate quantification of narrow chromatographic peaks. For co-eluting metabolites, compound specific precursor ions and their corresponding fragment ions were used for selective detection and quantification of those compounds. For instance, for 11,12-EpETE (m/z 317→195) and 12-HETE (m/z 319→135), both elute at RT 16.14 min. 

Compounds were separated using a Waters Acquity UPLC CSH C18 column (100 mm length × 2.1 mm id; 1.7 μm particle size) with an additional Waters Acquity VanGuard CSH C18 pre-column (5 mm × 2.1 mm id; 1.7 μm particle size) held constant at 60 °C. The mobile phase and gradient elution conditions were adopted from Pedersen and Newman [85]. In summary, the mobile phase consisted of (A) water (0.1% acetic acid) and (B) acetonitrile/isopropanol (ACN/IPA) (90/10, *v*/*v*) (0.1% acetic acid). Gradient elution conditions were carried out for 22 min at a flow rate of 0.15 mL min^−1^. Gradient conditions were: 0–1.0 min, 0.1–25% B; 1.0–2.5 min, 25–40% B; 2.5–4.5 min, 40–42% B; 4.5–10.5 min, 42–50% B; 10.5–12.5 min, 50–65% B; 12.5–14 min, 65–75% B; 14–14.5 min, 75–85% B; 14.5–20 min, 85–95% B; 20–20.5 min, 95–95% B; 20.5–22 min, 95–25% B. A 5 μL aliquot of each sample was injected onto the column. Limits of detection (LOD) and quantification (LOQ) (Table S3) were calculated based on one concentration point (0.1 ng µL^−1^) for each oxylipin and deuterated surrogate.

### 4.6. Data Processing 

#### 4.6.1. Untargeted Lipidomics

MS-DIAL (v. 2.80) was the software program used for data processing [89]. This open-source software permits processing of LC–MS data acquired either in MS^1^ only or with accompanying MS/MS information collected in data-dependent or data-independent mode from different MS platforms. We used LipidBlast [90] for lipid identification. Chromatographic peaks were annotated based on different levels of identification [91]. Peak intensities were normalized using the internal standard CUDA and the QC pool sample to correct for differences in injection volume and platform stability throughout the fully randomized batch of samples. The SPLASH Lipidomics Mix was used for the precise identification of major lipid classes and to perform relative quantitation.

#### 4.6.2. Targeted Analysis of Oxylipins 

Oxylipin data obtained by HPLC-dMRM-based analyses was processed using our in-house library on MultiQuant™ software. 

#### 4.6.3. Statistical Analyses

Annotated metabolites were used for multivariate statistical analysis. Pathway analysis, principal component analysis (PCA) and heat map plots were generated with MetaboAnalyst 4.0 [66]. The significance of individual metabolites between the treatment groups was assessed with a one-way ANOVA followed by Fisher’s post hoc analysis and Holm FDR-correction, with a *q*-value of <0.05 indicating significance. If needed, data was logarithmically transformed to correct for unequal variance or non-normal distribution. No outliers were excluded from the statistical analyses. Differences in oxylipins among treatments were analyzed in GraphPad Prism 7.03 (La Jolla, CA, USA). Discovery was determined using the two-stage linear step-up procedure of Benjamini et al., [92], with *q*-value = 5% (cutoff for FDR = 0.05). Each compound was analyzed individually, without assuming a consistent standard deviation. Figures were generated with GraphPad Prism 7.03 (La Jolla, CA), PowerPoint 2018 (Microsoft, Redmond, WA, USA), and MetaboAnalyst 4.0 [66].

## Figures and Tables

**Table 1:** Top 10 hepatic lipids positively and negatively associated with hepatic gene expression markers of inflammation ^1^.

Gene		Opn			Mcp1			CD68	
Association	Lipid	CC ^2^	*q*-Value ^3^	Lipid	CC	*q*-Value	Lipid	CC	*q*-Value
Positive	PG 38:4 18:1/20:3,ω6	0.74	5.9 × 10^−4^	Lyso PC 18:1	0.86	8.6 × 10^−6^	Lyso PC 18:1	0.78	5.3 × 10^−4^
Positive	Lyso PC 22:4,ω6	0.74	6.4 × 10^−4^	PC 17:0e 14:0e/3:0	0.85	1.3 × 10^−5^	SM d36:2 d14:0/22:2	0.78	5.4 × 10^−4^
Positive	Lyso PE 22:4,ω6	0.74	6.6 × 10^−4^	PC 16:0e 14:0e/2:0	0.84	2.3 × 10^−5^	Lyso PC 20:3,ω6	0.77	6.4 × 10^−4^
Positive	PG 36:4 16:0/20:4,ω6	0.73	8.4 × 10^−4^	SM d35:1 d14:0/21:1	0.82	3.8 × 10^−5^	PS 42:3 26:0/16:3	0.76	6.9 × 10^−4^
Positive	PG 38:5 18:1/20:4,ω6	0.72	9.9 × 10^−4^	PA 36:2 18:1/18:1	0.82	4.3 × 10^−5^	PC 17:0e 14:0e/3:0	0.76	7.7 × 10^−4^
Positive	20:1,ω9	0.72	1.1 × 10^−3^	PC 21:3e 18:3e/3:0	0.81	6.6 × 10^−5^	PC 21:3e 18:3e/3:0	0.74	1.0 × 10^−3^
Positive	PG 38:2 18:1/20:1	0.71	1.2 × 10^−3^	PC 18:1e 16:1e/2:0	0.81	6.7 × 10^−5^	SM d34:0 d14:0/20:0	0.74	1.1 × 10^−3^
Positive	PG 36:2 18:1/18:1	0.71	1.3 × 10^−3^	PE 20:1e 14:1e/6:0	0.80	7.8 × 10^−5^	PC 18:1e 16:1e/2:0	0.72	1.4 × 10^−3^
Positive	PG 42:9 20:3,ω6/22:6,ω3	0.71	1.3 × 10^−3^	PC 19:1e 14:1e/5:0	0.80	8.6 × 10^−5^	PC 16:0e 14:0e/2:0	0.72	1.4 × 10^−3^
Positive	Lyso PE 20:3,ω6	0.70	1.4 × 10^−3^	SM d34:1 d14:0/20:1	0.80	9.2 × 10^−5^	PE 20:1e 14:1e/6:0	0.72	1.6 × 10^−3^
Negative	PA 34:3 16:1,ω7/18:2,ω6	−0.69	1.9 × 10^−3^	12,13-DiHOME	−0.78	1.3 × 10^−4^	PI 36:2 18:1/18:1	−0.77	6.4 × 10^−4^
Negative	7,8-DiHDPE	−0.62	6.9 × 10^−3^	18:3,ω3	−0.76	2.9 × 10^−4^	PE 34:2 16:0/18:2,ω6	−0.76	6.9 × 10^−4^
Negative	13,14-DiHDPE	−0.62	6.9 × 10^−3^	PI 36:2 18:1/18:1	−0.76	2.9 × 10^−4^	PI 36:4 16:0/20:4,ω6	−0.76	7.7 × 10^−4^
Negative	7,8-EpDPE	−0.62	6.9 × 10^−3^	PE 40:6e 18:1e/22:5,ω3	−0.74	4.9 × 10^−4^	PI 36:3 16:0/20:3,ω6	−0.74	1.0 × 10^−3^
Negative	PE 34:2 16:0/18:2,ω6	−0.62	7.2 × 10^−3^	PI 36:4 16:0/20:4.ω6	−0.73	6.0 × 10^−4^	PE 34:3e 16:1e/18:2,ω3	−0.74	1.2 × 10^−3^
Negative	10,11-DiHDPE	−0.62	7.2 × 10^−3^	PE 34:2 16:0/18:2,ω6	−0.73	6.2 × 10^−4^	12,13-DiHOME	−0.73	1.4 × 10^−3^
Negative	Lyso PE 18:2,ω6	−0.62	7.4 × 10^−3^	18:2,ω6	−0.73	6.3 × 10^−4^	PE 36:2 18:0/18:2,ω6	−0.72	1.4 × 10^−3^
Negative	PI 36:3 16:0/20:3,ω6	−0.61	8.9 × 10^−3^	PA 34:3 16:1,ω7/18:2,ω6	−0.72	6.8 × 10^−4^	PE 36:2 18:1/18:1	−0.72	1.5 × 10^−3^
Negative	PC 35:2 13:1/22:1	−0.59	1.1 × 10^−2^	14,15-DiHETrE	−0.72	6.9 × 10^−4^	PE 40:6e 18:1e/22:5,ω3	−0.71	1.8 × 10^−3^
Negative	5,6-DiHETE	−0.59	1.1 × 10^−2^	13(S)-HODE	−0.71	1.1 × 10^−3^	18:3,ω3	−0.71	1.8 × 10^−3^

^1^ Associations between NASH inflammation markers and lipids were determined using the statistical package “Pattern Hunter in Metabolanalyst [66]. ^2^ CC, correlation coefficient; ^3^ *q*-value, see Statistical Analysis, Materials and methods.

**Table 2:** Associations between hepatic gene expression markers of fibrosis and lipids ^1^.

GENE		Col1A2			Timp1			Lox	
Association	Lipid	CC ^2^	*q*-Value ^3^	Lipid	CC	*q*-Value	Lipid	CC	*q*-Value
Positive	20:1,ω9	0.81	4.2 × 10^−5^	20:1,ω9	0.81	5.6 × 10^−5^	Lyso PC 22:4,ω6	0.84	1.2 × 10^−5^
Positive	18:1,ω9	0.81	4.9 × 10^−5^	18:1,ω7	0.80	9.0 × 10^−5^	PC 38:4 18:1/20:3,ω6	0.82	2.9 × 10^−5^
Positive	18:1,ω7	0.80	6.4 × 10^−5^	18:1,ω9	0.79	1.1 × 10^−4^	Lyso PE 22:4,ω6	0.80	5.3 × 10^−5^
Positive	Lyso PC 20:3,ω6	0.74	5.3 × 10^−4^	Lyso PC 20:3,ω6	0.74	6.7 × 10^−4^	PG 38:4 18:1/20:3,ω6	0.80	5.6 × 10^−5^
Positive	Lyso PE 20:3,ω6	0.73	6.7 × 10^−4^	16:0	0.73	8.1 × 10^−4^	PG 38:5 18:1/20:4,ω6	0.79	7.0 × 10^−5^
Positive	PC 38:4 18:1/20:3,ω6	0.72	1.1 × 10^−3^	Lyso PE 20:3, ω6	0.72	9.7 × 10^−4^	PG 38:1 20:0/18:1	0.79	7.0 × 10^−5^
Positive	Lyso PC 18:1,ω9	0.71	1.2 × 10^−3^	PC 38:4 18:1/20:3,ω6	0.71	1.3 × 10^−3^	PG 40:6 18:1/22:5,ω6	0.78	1.1 × 10^−4^
Positive	SM d35:1 d14:0/21:1	0.71	1.2 × 10^−3^	PC 21:3e 18:3e/3:0	0.71	1.3 × 10^−3^	PG 38:2 18:1/20:1	0.78	1.1 × 10^−4^
Positive	SM d36:2 d14:0/22:2	0.70	1.4 × 10^−3^	Lyso PC 18:1,ω9	0.71	1.3 × 10^−3^	PG 40:5 18:1/22:4,ω6	0.78	1.1 × 10^−4^
Positive	PA 36:2 18:1/18:1	0.70	1.4 × 10^−3^	SM d36:2 d14:0/22:2	0.69	2.2 × 10^−3^	PG 36:2 18:1/18:1	0.77	1.5 × 10^−4^
Negative	PA 34:3 16:1,ω7/18:2,ω6	−0.77	1.9 × 10^−4^	PA 34:3 16:1,ω7/18:2,ω6	−0.75	4.8 × 10^−4^	Lyso PE 18:2,ω6	−0.57	1.3 × 10^−2^
Negative	PE 34:2 16:0/18:2,ω6	−0.75	3.7 × 10^−4^	PE 34:2 16:0/18:2,ω6	0.73	7.1 × 10^−4^	8,9-DiHETE	−0.56	1.5 × 10^−2^
Negative	PI 36:3 16:0/20:3,ω6	−0.73	7.2 × 10^−4^	PI 36:3 16:0/20:3,ω6	−0.71	1.2 × 10^−3^	11,12-DiHETE	−0.56	1.5 × 10^−2^
Negative	PI 36:2 18:1/18:1	−0.72	8.7 × 10^−4^	PI 36:2 18:1/18:1	−0.71	1.3 × 10^−3^	8,9-EpETE	−0.56	1.5 × 10^−2^
Negative	PC 35:2 13:1/22:1	−0.71	1.1 × 10^−3^	PI 36:4 16:0/20:4,ω6	−0.69	1.9 × 10^−3^	14,15-EpETE	−0.56	1.7 × 10^−2^
Negative	PI 36:4 16:0/20:4,ω6	−0.71	1.1 × 10^−3^	PC 35:2 13:1/22:1	−0.69	2.0 × 10^−3^	17,18-DiHETE	−0.54	2.0 × 10^−2^
Negative	PE 36:2 18:1/18:1	−0.70	1.5 × 10^−3^	PE 36:2 18:1/18:1	−0.69	2.2 × 10^−3^	20:5,ω3	−0.54	2.2 × 10^−2^
Negative	PE 36:2 18:0/18:2,ω6	−0.68	2.4 × 10^−3^	PE 36:2 18:0/18:2,ω6	−0.66	3.7 × 10^−3^	PI 36:4 16:0/20:4,ω6	−0.53	2.3 × 10^−2^
Negative	PA 34:2 16:0/18:2,ω6	−0.66	3.5 × 10^−3^	10,11-DiHDPE	−0.65	4.3 × 10^−3^	PS 40:6 18:0/22:6,ω3	−0.53	2.3 × 10^−2^
Negative	7,8-DiHDPE	−0.66	3.8 × 10^−3^	PE 34:3e 16:1,ω7e/18:2,ω6	−0.65	4.4 × 10^−3^	10,11-EpDPE	−0.52	2.6 × 10^−2^

^1^ Associations between NASH inflammation markers and lipids were determined using the statistical package “Pattern Hunter in Metabolanalyst [66]. ^2^ CC, correlation coefficient; ^3^ *q*-value, see Statistical Analysis, Materials and methods.

## Supplementary Materials

The following are available online at https://www.mdpi.com/2218-1989/9/11/252/s1, Figure S1. LC-MS/MS chromatogram of 60 transitions in a 22 min LC-run allowing monitoring 39 oxylipins, 17 deuterated oxylipins, CUDA, and the deuterated surrogates eicosapentaenoic acid-d5 (EPA-d5), docosahexaenoic acid-d5 (DHA-d5), and arachidonic acid-d8 (ARA-d8). Analysis were performed on a SCIEX linear ion trap (LIT) QTRAP 4000 using the dMRM method implemented from Pedersen et al., [80]. The use of a quadrupole mass spectrometer with a linear ion trap significantly enhances platform performance by increasing ion capacity, improving injection and trapping efficiencies, and increasing duty cycle, Table S1. Diet effects on all lipids, Table S2. Lipids significantly affected by diet, Table S3. Detailed list of multi-reaction monitoring (MRM) transitions for the deuterated-oxylipins (surrogates) and CUDA (12-[[(cyclohexylamino) carbonyl] amino]-dodecanoic acid) used as internal standards for our analysis. Compounds are ordered based on retention time (RT), Table S4. Detailed list of multi-reaction monitoring (MRM) transitions for the oxylipins contained in our in-house library. Compounds are ordered based on retention time (RT).
