# Supplementary material for: A Lipidomic Analysis of Docosahexaenoic Acid (22:6, ω3) Mediated Attenuation of Western Diet Induced Nonalcoholic Steatohepatitis in Male Ldlr -/- Mice
Source: Metabolites. 2019 Oct 28;9(11):252. doi: 10.3390/metabo9110252 (PMC6918288; doi:10.3390/metabo9110252)
Supplement: Supplementary file 1 [file metabolites-09-00252-s001.zip › Supplementary Materials.pdf]

Supplementary Materials

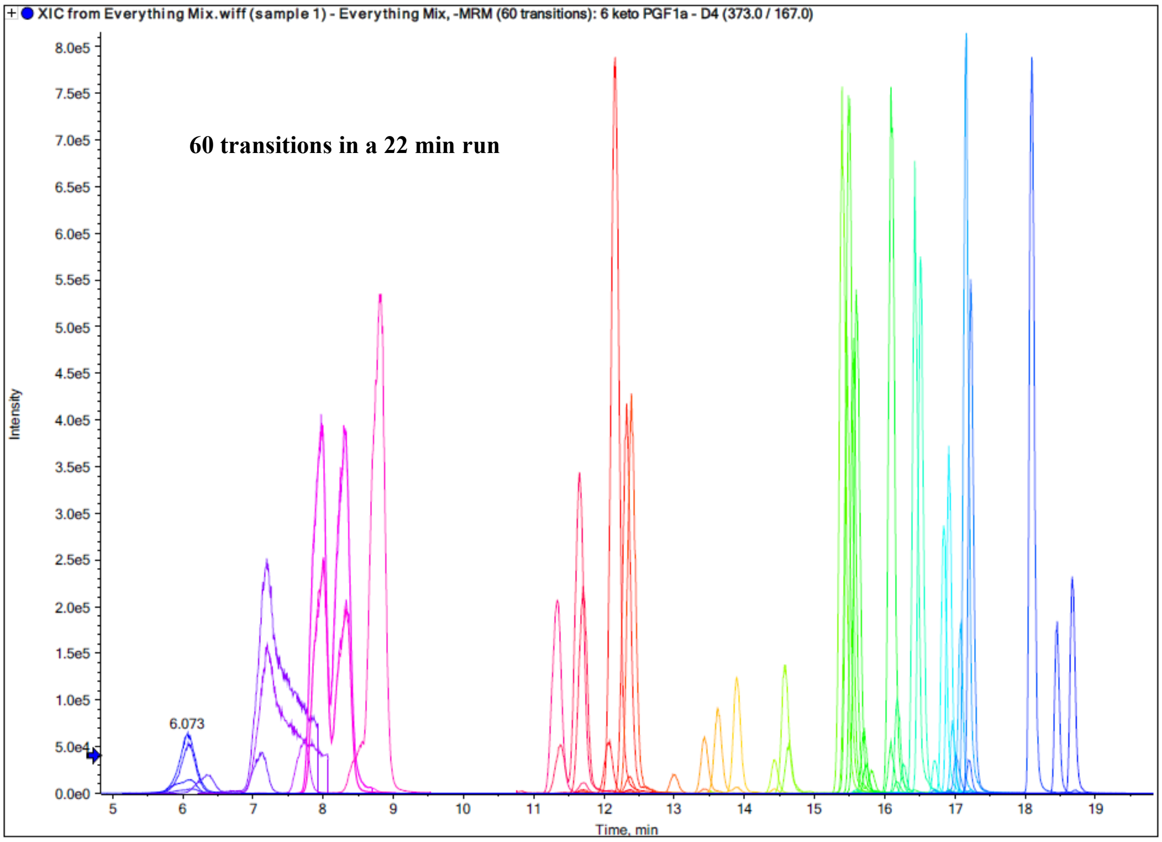

Figure 1. LC-MS/MS chromatogram of 60 transitions in a 22 min LC-run allowing monitoring 39 oxylipins, 17 deuterated oxylipins, CUDA, and the deuterated surrogates eicosapentaenoic acid-d5 (EPA-d5), docosahexaenoic acid-d5 (DHA-d5), and arachidonic acid-d8 (ARA-d8). Analysis were performed on a SCIEX linear ion trap (LIT) QTRAP 4000 using the dMRM method implemented from Pedersen et al., [80]. The use of a quadrupole mass spectrometer with a linear ion trap significantly enhances platform performance by increasing ion capacity, improving injection and trapping efficiencies, and increasing duty cycle.

Table S1. Diet effects on all lipids.See zip file

Table S2. Lipids significantly affected by diet. See zip file

Table S3. Detailed list of multi-reaction monitoring (MRM) transitions for the deuterated-oxylipins (surrogates) and CUDA (12-[[(cyclohexylamino) carbonyl] amino]-dodecanoic acid) used as internal standards for our analysis. Compounds are ordered based on retention time (RT).

Surrogates	Precurson Ion	Product Ion	RT	DP	CE	S/N	LOD (ng/µl)	LOQ (ng/µl)
**6 keto PGF1α - d4**	373	167	6.0	-70	-40	5.7	0.084	0.281
**Resolvin E1 - d4**	353	197	6.0	-40	-20	11.4	0.042	0.140
**Thromboxane B2 - d4**	373	173	7.1	-50	-21	24.6	0.020	0.065
**PGF2a - d4**	357	197	7.6	-70	-33	2.8	0.171	0.571
**PGE2 - d4**	355	275	7.9	-35	-25	43.6	0.011	0.037
**PGD2 - d4**	355	275	8.2	-35	-25	65.3	0.007	0.025
**Resolvin D1 - d5**	380	141	8.7	-40	-20	1.5	0.320	1.067
**Leukotriene B4 - d4**	339	197	11.6	-70	-21	60.8	0.008	0.026
**CUDA**	339	214	12.1	-65	-35	1310.3	0.000	0.001
**12,13-DiHOME - d4**	317	185	12.3	-70	-30	52.3	0.009	0.031
**20-HETE - d6**	325	281	14.5	-65	-24	3.1	0.155	0.516
**13(S)-HODE - d4**	299	198	15.3	-65	-25	30.5	0.016	0.053
**9(S)-HODE - d4**	299	172	15.4	-60	-25	5.3	0.091	0.302
**15(S)-HETE - d8**	327	226	15.5	-70	-16	63602.5	0.000	0.000
**12(S)-HETE - d8**	327	184	16.0	-60	-21	15	0.032	0.107
**5(S)-HETE - d8**	327	116	16.4	-50	-20	35.6	0.014	0.045
**14,15 - EET(EpETrE) - d11**	330	175	16.8	-70	-16	48.3	0.010	0.033
**11,12-EET (EpETrE)- d11**	330	167	17.1	-55	-15	8.8	0.055	0.182
**EPA - d5**	306	262	18.1	-55	-20	5.4	0.089	0.296
**DHA - d5**	332	234	18.4	-55	-20	10.5	0.046	0.152
**ARA - d8**	311	267	18.6	-60	-18	15.2	0.032	0.105

RT: retention time (min); DP: declustering potential (V); CE: collision energy (V); S/N: Signal to noise ratio; LOD: limit of detection; LOQ: limit of quantification.

Table 4. Detailed list of multi-reaction monitoring (MRM) transitions for the oxylipins contained in our *in-house* library. Compounds are ordered based on retention time (RT).

Precursor	Pathway	Compound	Precurson Ion	Product Ion	RT	DP	CE	S/N	LOD (ng/µl)	LOQ (ng/µl)
C20:4	COX	6-keto PGF1α	369	163	6.0	-70	-40	21.3	0.023	0.075
C20:5	COX	Resolvin E1	349	195	6.1	-40	-20	6.7	0.072	0.239
C20:5	ROS	8-iso PGF3α	351	307	6.3	-80	-26	11.4	0.042	0.140
C20:4	COX	8-iso PGF2α	353	193	7.1	-70	-33	5.9	0.081	0.271
C20:4	COX	Thromboxane B2	369	169	7.2	-50	-21	309.7	0.002	0.005
C20:4	COX	PGE2	351	271	7.9	-35	-25	651.8	0.001	0.003
C20:4	COX	PGD2	351	271	8.3	-35	-25	505.8	0.001	0.003
C22:6	LOX	Resolvin D1	375	121	8.8	-40	-20	10	0.048	0.160
C22:6	LOX	PDX	359	153	11.3	-20	-20	6186.1	0.000	0.000
C20:5	CYPEPOX/sEH	17,18-DiHETE	335	203	11.4	-60	-22	3689.7	0.000	0.000
C20:4	LOX5	Leukotriene B4	335	195	11.6	-70	-21	2163.1	0.000	0.001
C20:5	CYPEPOX/sEH	14,15-DiHETE	335	111	11.8	-55	-22	810.4	0.001	0.002
C20:5	CYPEPOX/sEH	11,12-DiHETE	335	167	12.0	-55	-22	1380.4	0.000	0.001
C20:5	CYPEPOX/sEH	8,9-DiHETE	335	185	12.3	-55	-22	7	0.069	0.229
C18:2	CYPEPOX/sEH	12,13-DiHOME	313	183	12.3	-70	-30	1262.4	0.000	0.001
C20:5	CYPEPOX/sEH	5,6-DiHETE	335	145	12.9	-55	-22	17.7	0.027	0.090
C22:6	CYPEPOX/sEH	19,20-DiHDPA	361	229	12.9	-74	-24	1.3	0.369	1.231
C20:4	CYPEPOX/sEH	14,15-DiHET	337	207	13.0	-65	-25	38.8	0.012	0.041
C22:6	CYPEPOX/sEH	16,17-DiHDPA	361	233	13.3	-80	-24	8.6	0.056	0.186
C22:6	CYPEPOX/sEH	13,14-DiHDPA	361	193	13.6	-80	-24	39.9	0.012	0.040
C22:6	CYPEPOX/sEH	10,11-DiHDPA	361	153	13.8	-80	-24	23.4	0.021	0.068
C22:6	CYPEPOX/sEH	7,8-DiHDPA	361	127	14.4	-80	-24	25.8	0.019	0.062
C20:4	CYPOH	20-HETE	319	245	14.6	-65	-24	42.7	0.011	0.038
C18:2	LOX12/15	13(S)-HODE	295	195	15.4	-65	-25	2871	0.000	0.001
C18:2	LOX5	9(S)-HODE	295	171	15.5	-60	-25	2743.5	0.000	0.001
C20:4	LOX12/15	15-HETE	319	175	15.7	-70	-16	8.7	0.055	0.184
C20:5	CYPEPOX	17,18-EpETE	317	215	15.7	-55	-15	34.8	0.014	0.046
C20:5	CYPEPOX	14,15-EpETE	317	248	16.0	-45	-15	7754.4	0.000	0.000
C20:5	CYPEPOX	11,12-EpETE	317	195	16.1	-70	-16	17.3	0.028	0.093
C20:4	LOX12/15	12-HETE	319	135	16.1	-60	-21	50.3	0.010	0.032
C20:5	CYPEPOX	8,9-EpETE	317	155	16.3	-75	-16	3721.6	0.000	0.000
C20:4	LOX5	5-HETE	319	115	16.5	-50	-20	80.6	0.006	0.020
C22:6	CYPEPOX	19,20-EpDPA	343	241	16.7	-45	-20	11.4	0.042	0.140
C20:4	CYPEPOX	14,15-EET	319	175	16.9	-70	-16	1924.7	0.000	0.001
C22:6	CYPEPOX	16,17-EpDPA	343	274	16.9	-55	-15	55832.9	0.000	0.000
C22:6	CYPEPOX	10,11-EpDPA	343	153	17.0	-55	-15	152.8	0.003	0.011
C22:6	CYPEPOX	13,14-EpDPA	343	161	17.2	-55	-15	2.5	0.192	0.640
C20:4	CYPEPOX	11,12-EET	319	167	17.2	-55	-15	985.6	0.001	0.002
C22:6	CYPEPOX	7,8-EpDPA	343	113	17.2	-55	-15	11.5	0.042	0.139

COX: cyclooxygenases; LOX: lipoxygenases; ROS: reactive oxygen species; CYPEPOX/sEH: cytochrome P450 epoxide/soluble epoxy hydrolase; CYPOH: CYP omega hydroxylases; RT: retention time (min); DP: declustering potential (V); CE: collision energy (V); S/N: Signal to noise ratio; LOD: limit of detection; LOQ: limit of quantification.
