# Supplementary material for: A Lipidomic Analysis of Docosahexaenoic Acid (22:6, ω3) Mediated Attenuation of Western Diet Induced Nonalcoholic Steatohepatitis in Male Ldlr -/- Mice
Source: Metabolites. 2019 Oct 28;9(11):252. doi: 10.3390/metabo9110252 (PMC6918288; doi:10.3390/metabo9110252)
Supplement: Supplementary file 1 [file metabolites-09-00252-s001.zip › SWATH parameters_SI.pdf]

SWATH parameters for un-targeted analysis

For ESI(+), the SWATH parameters were MS^1 accumulation time, 100 ms; MS^1 mass range, m/z 100–1700; MS^2 accumulation time, 10 ms; collision energy, 45 eV; collision energy spread, 15 eV; cycle time, 550 ms; Q_1 window, 20 Da; SWATH mass range, m/z 300–1100; number of SWATH experiments, 40; MS^2 mass range: m/z 100–1100. Other parameters were curtain gas, 35; ion source gas 1, 60; ion source gas 2, 60; temperature, 350°C; ion spray voltage floating, 4.5 kV; declustering potential, 80 V. For ESI(–), the SWATH parameters were MS^1 accumulation time, 100 ms; MS^1 mass range, m/z 100–1700; MS^2 accumulation time, 10 ms; collision energy, -45 eV; collision energy spread, 15 eV; cycle time, 550 ms; Q_1 window, 20 Da; SWATH mass range, m/z 220–1020; number of SWATH experiments, 40; MS^2 mass range: m/z 100–1000. Other parameters were curtain gas, 35; ion source gas 1, 60; ion source gas 2, 60; temperature, 350°C; ion spray voltage floating, -4.5 kV; declustering potential, -80 V.
